# The role of hand preference in cognition and neuropsychiatric symptoms in neurodegenerative diseases

**DOI:** 10.1093/braincomms/fcad137

**Published:** 2023-04-24

**Authors:** Toni T Saari, Eero Vuoksimaa

**Affiliations:** Brain Research Unit, Department of Neurology, University of Eastern Finland, Kuopio 70210, Finland; Department of Neurology, NeuroCenter, Kuopio University Hospital, Kuopio 70210, Finland; Institute for Molecular Medicine Finland (FIMM), HiLIFE, University of Helsinki, Helsinki 00290, Finland; Institute for Molecular Medicine Finland (FIMM), HiLIFE, University of Helsinki, Helsinki 00290, Finland

**Keywords:** handedness, dementia, neurodegenerative diseases, cognition, neuropsychiatric symptoms

## Abstract

Handedness has been shown to be associated with genetic variation involving brain development and neuropsychiatric diseases. Whether handedness plays a role in clinical phenotypes of common neurodegenerative diseases has not been extensively studied. This study used the National Alzheimer’s Coordinating Center database to examine whether self-reported handedness was associated with neuropsychological performance and neuropsychiatric symptoms in cognitively unimpaired individuals (*n* = 17 670), individuals with Alzheimer’s disease (*n* = 10 709), behavioural variant frontotemporal dementia (*n* = 1132) or dementia with Lewy bodies (*n* = 637). Of the sample, 8% were left-handed, and 2% were ambidextrous. There were small differences in the handedness distributions across the cognitively unimpaired, Alzheimer’s disease, behavioural variant frontotemporal dementia and dementia with Lewy bodies groups (7.2–9.5% left-handed and 0.9–2.2% ambidextrous). After adjusting for age, gender and education, we found faster performance in Trail Making Test A in cognitively unimpaired non-right-handers (ambidextrous and left-handed) compared with right-handers. Excluding ambidextrous individuals, the left-handed cognitively unimpaired individuals had faster Trail Making Test A performance and better Number Span Forward performance than right-handers. Overall, handedness had no effects on most neuropsychological tests and none on neuropsychiatric symptoms. Handedness effect on Trail Making Test A in the cognitively unimpaired is likely to stem from test artefacts rather than a robust difference in cognitive performance. In conclusion, handedness does not appear to affect neuropsychological performance or neuropsychiatric symptoms in common neurodegenerative diseases.

## Teaser

Saari and Vuoksimaa investigate whether hand preference associates with cognitive test performance or neuropsychiatric symptoms in Alzheimer’s disease, behavioural variant frontotemporal dementia, dementia with Lewy bodies or normal cognitive aging using a large data set. The researchers find no robust handedness-related differences in cognitive performance or neuropsychiatric symptoms in any groups.

## Graphical Abstract

**Graphical Abstract:**
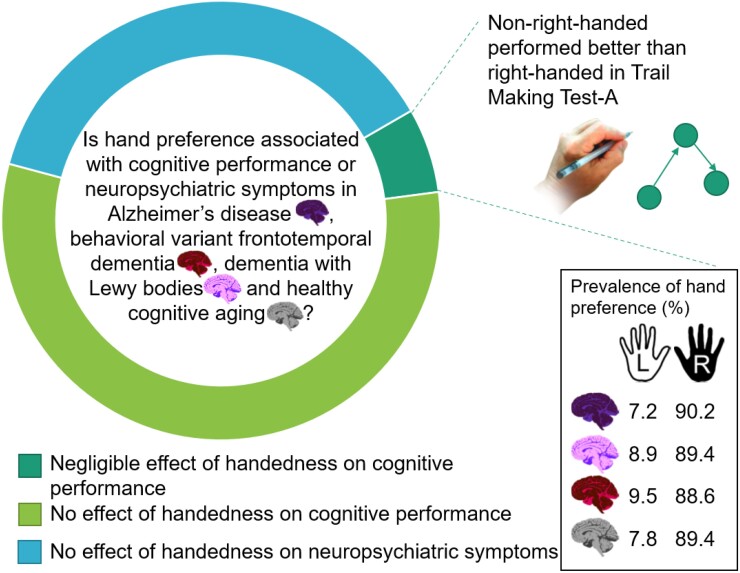

See McManus (https://doi.org/10.1093/braincomms/fcad162) for a scientific commentary on this article.

## Introduction

Recent studies have found associations between genetic variants associated with handedness and neuropsychiatric diseases, such as schizophrenia.^1,2^ Moreover, genetic factors in handedness also associate with brain development and morphology, possibly relating to selective vulnerability to neurodegenerative diseases in late life.^3,4^ There is also an increased prevalence of non-right-handedness in schizophrenia^5^ and bipolar disorder.^6^ However, in contrast to early hypotheses emphasizing brain asymmetry as relevant to pathogenesis of schizophrenia, a large study found no evidence for handedness–cognition relationships in patients with schizophrenia.^7^

Relatively few studies exist concerning the role of handedness in neurodegenerative diseases. In primary progressive aphasia, non-right-handedness was over-represented in the semantic variant of the disease, yet no cognitive differences emerged between handedness groups.^4^ An early study in Alzheimer’s disease found that a small group of left-handed patients did not differ in language skills, visuospatial abilities or motor function compared with right-handed patients matched for global cognition, age and education.^8^ More recently, a review suggested that core symptoms of neurodegenerative diseases may manifest asymmetrically,^3^ and recent studies have found evidence of atypical variants of behavioural variant frontotemporal dementia (bvFTD)^9^ and Alzheimer’s disease that primarily affect the right hemisphere.^10^ Together, these findings suggest that the pathology and accompanying symptoms can be clearly asymmetrical in common neurodegenerative diseases. However, whether handedness as a proxy of brain asymmetry is related to clinical differences in common neurodegenerative diseases has not been thoroughly examined. Answering this question requires an extensive sample size, and the goal of this study is to leverage a large data set to explore whether handedness plays a role in neuropsychological or neuropsychiatric functioning in common neurodegenerative diseases.

## Materials and methods

### Study participants

This study used data from the University of Washington’s National Alzheimer Coordinating Data Center (NACC) Uniform Data Set (UDS). Study participants underwent standardized neuropsychological assessment and behavioural evaluation. Study participants and informants gave written informed consent. Individuals with no diagnosed cognitive impairment formed the cognitively unimpaired (CU) group. In order to study the role of handedness for separate neurodegenerative diseases, we excluded cases with significant vascular comorbidities and those with multiple diagnoses [e.g. Alzheimer’s disease + dementia with Lewy bodies (DLB)]. For the DLB group, those with a suspected Parkinson’s disease dementia were excluded. The data were collected between 2005 and 2022, and clinical diagnoses were made using available diagnostic criteria.^11-17^ Slightly different cognitive measures have been used in the UDS versions 2 and 3.

### Neuropsychological and neuropsychiatric measures

UDS versions 1 and 2 differ in respect to some clarifications in instructions and additional scoring of error types,^18^ not in terms of cognitive measures included. Thus, consistent with recent publications on the NACC data (e.g. Dodge *et al*.^19^), we treat version 1 and 2 data equally (later; version 2). UDS version 2 measures included Mini-Mental State Examination^20^ as a cognitive screening instrument. Specific cognitive domains were measured with Digit Span Forward^21^ as a measure of attention, Digit Symbol^22^ and Trail Making Test (TMT) A^23^ as measures of visuomotor processing speed, Digit Span Backward and TMT-B as measures of executive functions and Logical Memory Story A Immediate and Delayed Recall^21^ as measures of episodic memory. Language skills were assessed with animal and vegetable fluency and Boston Naming Test.^24^ UDS version 3 was implemented in 2015 and used Montreal Cognitive Assessment^25^ as a cognitive screening instrument. In UDS version 3, Logical Memory story was replaced by Craft story task,^26^ Digit Span tests were replaced by Number Span tests, Boston Naming Test was replaced by Multilingual Naming Test,^27^ and Benson Complex Figure^28^ was added as a measure of visuoconstructional ability and delayed recall of visual material. Neuropsychiatric symptoms were assessed with the Neuropsychiatric Inventory Questionnaire^29^ at all visits. Clinical Dementia Rating (CDR) Dementia Staging Instrument was used to describe the disease severity in the clinical groups.^30^

### Handedness measure

Hand preference (hereafter handedness) was measured with a self-report (at the time of study visit) of being left-handed, right-handed or ambidextrous. In our analyses, we combined left-handed and ambidextrous individuals into one group of non-right-handedness in line with previous research.^4,6,7^ However, as most of self-reported ambidextrous individuals write with their right rather than left hand^31^ and because ambidextrous individuals are genetically distinct from left-handers,^1^ we also ran our main analyses by contrasting left- and right-handers by excluding ambidextrous individuals.

### Statistical analyses

Statistical analyses were conducted in R version 4.2.1.^32^ The code for all analyses can be found online (https://osf.io/38qj6/). Differences between clinical and handedness groups were analysed using one-way analysis of variance with Tukey’s *post hoc* test, two-tailed *t*-tests, chi-square tests and Fisher’s exact tests. Multiple regression analyses were conducted using neuropsychological and neuropsychiatric variables as dependent variables and age, gender, education and handedness as independent variables. Adjustments for multiple comparisons were made using Bonferroni–Holm method.^33^ Results were considered statistically significant at *α* < 0.05.

## Results

### Demographic statistics and handedness in neurodegenerative diseases

Handedness information was available for 99.3% (*n* = 30 148) of the participants; 0.7% (*n* = 212) with missing handedness information were excluded from the analyses. In the whole sample, 90.3% (*n* = 27 218) were right-handed, 7.8% (*n* = 2337) were left-handed, and 2% (*n* = 593) were ambidextrous. The demographical and clinical information of the study sample is presented in Table 1. The distribution of left-handed, right-handed and ambidextrous individuals differed between CU, Alzheimer’s disease, bvFTD and DLB groups (*P* < 0.001). When examining Pearson residuals (using a cut-off = |2|) of all possible combinations of handedness and disease groups, we found that there were more left-handed DLB individuals and more ambidextrous CU participants than expected, with fewer than expected ambidextrous individuals with Alzheimer’s disease. The effect, however, was very small (Cramér’s *V* = 0.02). The prevalence of left-handers varied from 7.2% to 9.5% in different groups (Table 1).

**Table 1:** Demographic characteristics of the study sample

	CU	Alzheimer’s disease	DLB	bvFTD
*N*	17 772	10 806	642	1140
Age, mean (SD)	70.14 (10.74)	74.01 (10.19)	72.83 (7.68)	62.82 (9.48)
Education, mean (SD)	15.79 (3)	14 (4.3)	14.85 (4.17)	15.24 (3.24)
Women, *n* (%)	11 600 (65.3%)	6120 (56.6%)	153 (23.8%)	449 (39.4%)
Race, *n* (%)				
Caucasian	14 039 (79%)	8840 (81.8%)	578 (90%)	1067 (93.6%)
African-American	2800 (15.76%)	1277 (11.8%)	34 (5.3%)	26 (2.3%)
American Indian or Alaska Native	148 (0.83%)	115 (1.1%)	11 (1.7%)	2 (0.2%)
Native Hawaiian or Pacific Islander	15 (0.08%)	11 (0.1%)	0	2 (0.2%)
Asian	515 (2.9%)	206 (1.9%)	10 (1.6%)	29 (2.5%)
Other	151 (0.85%)	291 (2.7%)	7 (1.1%)	5 (0.4%)
NA	104 (0.59%)	66 (0.6%)	2 (0.3%)	9 (0.8%)
Handedness, *n* (%)				
Left-handed	1391 (7.8%)	781 (7.2%)	57 (8.9%)	108 (9.5%)
Right-handed	15 885 (89.4%)	9749 (90.2%)	574 (89.4%)	1010 (88.6%)
Ambidextrous	394 (2.2%)	179 (1.7%)	6 (0.9%)	14 (1.2%)
Unknown	102 (0.6%)	97 (0.9%)	5 (0.8%)	8 (0.7%)
CDR Global Score, *n* (%)				
0.5		3396 (31.4%)	187 (29.1%)	274 (24%)
1		4867 (45%)	289 (45%)	494 (43.3%)
2		1674 (15.5%)	113 (17.6%)	229 (20.1%)
3		824 (7.6%)	52 (8.1%)	137 (12%)
NA		45 (0.4%)	1 (0.2%)	6 (0.5%)

CDR, Clinical Dementia Rating.

Men had higher prevalence of left-handedness (9%, *n* = 1073) than women (6.9%, *n* = 1264). Left-handed individuals were more educated than right-handers in CU [*F*(2,17577) = 13.11, *P* < 0.001, Tukey’s *post hoc* test *P* < 0.001] and Alzheimer’s disease groups [*F*(2,10625) = 15.72, *P* < 0.001, Tukey’s *post hoc* test *P* < 0.001]. Age differences were also observed in the CU group [*F*(2,17667) = 14.15, *P* < 0.001]: left-handers were younger than right-handers (*P* < 0.001) and ambidextrous individuals (*P* = 0.016). Similarly in the Alzheimer’s disease group, left-handers were younger than ambidextrous and right-handed individuals; additionally, ambidextrous individuals were younger than right-handers [*F*(2,10706) = 26.35, *P* = 3.86 × 10^−12^, all *post hoc P <* 0.05]. In analyses of gender and handedness distributions, CU men were more often ambidextrous or left-handed than expected [*χ*^2^(2) = 20.408, *P* < 0.001]. In the Alzheimer’s disease group, there were more left-handed men than expected [*χ*^2^(2) = 47.415, *P* < 0.001]. Only DLB group showed differences in CDR global score distributions by handedness (Fisher’s exact test, *P* = 0.032); however, the *post hoc* pairwise comparisons of handedness and CDR scores were not significant after adjusting for multiple comparisons.

### Associations of handedness with neuropsychological and neuropsychiatric measures

In line with previous research on handedness differences, left-handed and ambidextrous individuals were analysed together as a non-right-handed group. Tables 2–5 (see Supplementary Table 1 for sample and effect sizes per variable) show the comparison of right-handed and non-right-handed individuals’ neuropsychological performance and neuropsychiatric symptom ratings in all diagnostic groups.

**Table 2:** Comparison of neuropsychological test performance and neuropsychiatric symptoms between right-handed and non-right-handed CU individuals (*n* = 17 670)

	Non-right-handed		Right-handed		*P*
	Mean	SD	Mean	SD	
**Neuropsychological tests**					
Animal fluency	20.91	5.57	20.47	5.64	**0**.**002**
Vegetable fluency	14.75	4.24	14.66	4.25	0.405
F + L phonemic fluency	29.43	8.28	28.00	8.16	**0**.0001655*
TMT-A	31.52	14.44	34.42	16.13	**0**.00000000000001606*
TMT-B	83.15	45.08	90.19	50.63	**0**.000000002417*
MMSE^a^	28.95	1.38	28.84	1.46	**0**.**016**
Logical Memory Immediate^a^	13.54	3.71	13.38	3.89	0.154
Logical Memory Delayed^a^	12.24	4.00	12.10	4.19	0.256
Digit Span Forward^a^	8.68	2.09	8.49	2.08	**0**.**004**
Digit Span Backward^a^	6.94	2.20	6.74	2.25	**0**.**003**
Boston Naming Test^a^	27.38	2.98	26.93	3.44	**0**.000002167*
Digit Symbol^a^	48.03	12.38	47.17	12.99	**0**.**028**
MoCA^b^	26.22	2.87	26.11	2.84	0.384
Craft Story Immediate^b^	15.91	3.86	16.07	3.90	0.377
Craft Story Delayed^b^	14.87	4.22	14.96	4.13	0.650
Number Span Forward^b^	8.61	2.46	8.20	2.36	**0**.0002255*
Number Span Backward^b^	7.31	2.31	6.95	2.20	**0**.000682*
Multilingual Naming Test^b^	30.04	2.20	29.80	2.49	**0**.**016**
Copy of Benson Figure^b^	15.40	1.50	15.52	1.33	0.099
Benson Figure Delayed Recall^b^	11.30	3.06	11.42	2.87	0.405
**Neuropsychiatric symptoms**					
Delusions	0.02	0.17	0.01	0.12	0.179
Hallucinations	0.00	0.08	0.00	0.08	0.923
Agitation/aggression	0.07	0.30	0.07	0.33	0.380
Depression/dysphoria	0.16	0.47	0.18	0.49	0.200
Anxiety	0.14	0.44	0.13	0.43	0.287
Elation/euphoria	0.01	0.15	0.01	0.15	0.598
Apathy/indifference	0.05	0.27	0.06	0.31	0.111
Disinhibition	0.03	0.22	0.04	0.25	0.345
Irritability/lability	0.14	0.45	0.15	0.45	0.710
Motor disturbance	0.02	0.20	0.02	0.18	0.340
Nighttime behaviours	0.15	0.49	0.15	0.49	0.720
Appetite/eating	0.07	0.32	0.08	0.33	0.696

Numbers are raw scores in neuropsychological tests and the severity rating of Neuropsychiatric Inventory Questionnaire. Nominally significant *P*-values are bolded. MMSE, Mini-Mental State Examination; MoCA, Montreal Cognitive Assessment.

a UDS version 2 tests.

b UDS version 3 tests.

* Significant after Bonferroni–Holm adjustment for multiple comparisons.

**Table 3:** Comparison of neuropsychological test performance and neuropsychiatric symptoms between right-handed individuals and non-right-handed individuals with Alzheimer’s disease (*n* = 10 709)

	Non-right-handed		Right-handed		*P*
	Mean	SD	Mean	SD	
**Neuropsychological tests**					
Animal fluency	10.56	5.28	10.24	5.23	0.094
Vegetable fluency	6.49	3.78	6.64	3.90	0.280
F + L phonemic fluency	17.92	9.35	18.08	9.42	0.810
TMT-A	68.54	41.87	74.73	42.56	**0**.0001242*
TMT-B	208.73	90.17	215.20	87.46	0.104
MMSE^a^	20.12	6.70	19.52	6.68	**0**.**026**
Logical Memory Immediate^a^	4.12	3.64	3.89	3.46	0.134
Logical Memory Delayed^a^	1.88	2.97	1.81	2.89	0.602
Digit Span Forward^a^	6.81	2.41	6.63	2.37	0.078
Digit Span Backward^a^	4.36	2.12	4.18	2.09	**0**.**049**
Boston Naming Test^a^	19.62	7.29	18.55	7.64	**0**.**0006296**
Digit Symbol^a^	24.73	13.76	23.91	13.82	0.190
MoCA^b^	14.63	5.94	14.41	6.15	0.600
Craft Story Immediate^b^	6.13	4.16	6.38	4.35	0.399
Craft Story Delayed^b^	3.00	3.89	3.21	4.07	0.459
Number Span Forward^b^	6.38	2.36	6.19	2.44	0.260
Number Span Backward^b^	4.11	2.26	4.07	2.24	0.804
Multilingual Naming Test^b^	24.50	6.53	23.78	7.00	0.137
Copy of Benson Figure^b^	11.82	4.91	12.16	4.95	0.348
Benson Figure Delayed Recall^b^	2.56	3.52	2.80	3.57	0.357
**Neuropsychiatric symptoms**					
Delusions	0.25	0.66	0.28	0.68	0.291
Hallucinations	0.11	0.44	0.13	0.48	0.099
Agitation/aggression	0.52	0.82	0.53	0.83	0.597
Depression/dysphoria	0.60	0.81	0.60	0.83	0.964
Anxiety	0.69	0.89	0.62	0.86	**0**.**013**
Elation/euphoria	0.07	0.33	0.06	0.31	0.460
Apathy/indifference	0.72	0.93	0.69	0.92	0.352
Disinhibition	0.31	0.67	0.31	0.68	0.917
Irritability/lability	0.60	0.82	0.59	0.84	0.899
Motor disturbance	0.32	0.70	0.36	0.75	0.118
Nighttime behaviours	0.50	0.84	0.46	0.83	0.254
Appetite/eating	0.44	0.78	0.42	0.77	0.482

Numbers are raw scores in neuropsychological tests and the severity rating of Neuropsychiatric Inventory Questionnaire. Nominally significant *P*-values are bolded. MMSE, Mini-Mental State Examination; MoCA, Montreal Cognitive Assessment.

a UDS version 2 tests.

b UDS version 3 tests.

* Significant after Bonferroni–Holm adjustment for multiple comparisons.

**Table 4:** Comparison of neuropsychological test performance and neuropsychiatric symptoms between right-handed individuals and non-right-handed individuals with dementia with Lewy bodies (*n* = 637)

	Non-right-handed		Right-handed		*P*
	Mean	SD	Mean	SD	
**Neuropsychological tests**					
Animal fluency	11.02	5.23	10.60	5.24	0.597
Vegetable fluency	6.63	2.89	6.68	3.51	0.906
F + L phonemic fluency	16.06	8.85	16.70	8.53	0.776
TMT-A	90.53	45.26	97.32	43.35	0.341
TMT-B	258.35	70.74	248.89	74.56	0.522
MMSE^a^	20.09	6.29	21.07	6.38	0.403
Logical Memory Immediate^a^	5.20	3.39	5.91	3.99	0.282
Logical Memory Delayed^a^	3.87	4.05	4.30	3.88	0.576
Digit Span Forward^a^	6.10	1.95	6.65	2.18	0.148
Digit Span Backward^a^	3.59	1.82	3.87	1.87	0.429
Boston Naming Test^a^	22.50	5.32	22.11	6.31	0.707
Digit Symbol^a^	16.29	11.10	18.01	11.31	0.502
MoCA^b^	17.55	2.40	16.36	2.11	0.349
Craft Story Immediate^b^	8.67	5.09	8.62	4.65	0.968
Craft Story Delayed^b^	7.17	4.81	6.71	4.54	0.708
Number Span Forward^b^	6.42	1.84	6.50	2.52	0.871
Number Span Backward^b^	3.47	1.50	3.85	1.92	0.343
Multilingual Naming Test^b^	27.84	3.47	26.90	4.64	0.309
Copy of Benson Figure^b^	13.61	3.45	11.54	4.64	**0**.**033**
Benson Figure Delayed Recall^b^	8.65	4.40	5.42	3.59	**0**.**010**
**Neuropsychiatric symptoms**					
Delusions	0.50	0.79	0.53	0.90	0.764
Hallucinations	0.85	1.02	0.73	0.95	0.408
Agitation/aggression	0.58	0.91	0.60	0.88	0.890
Depression/dysphoria	0.69	0.79	0.78	0.88	0.426
Anxiety	1.02	0.95	0.93	0.99	0.517
Elation/euphoria	0.02	0.13	0.06	0.29	**0**.**023**
Apathy/indifference	0.80	0.97	1.09	1.05	**0**.**035**
Disinhibition	0.22	0.52	0.32	0.71	0.151
Irritability/lability	0.67	0.84	0.68	0.90	0.907
Motor disturbance	0.33	0.68	0.52	0.89	0.050
Nighttime behaviours	1.17	1.08	1.19	1.12	0.882
Appetite/eating	0.56	0.93	0.67	0.96	0.374

Numbers are raw scores in neuropsychological tests and the severity rating of Neuropsychiatric Inventory Questionnaire. Nominally significant *P*-values are bolded. MMSE, Mini-Mental State Examination; MoCA, Montreal Cognitive Assessment.

a UDS version 2 tests.

b UDS version 3 tests.

**Table 5:** Comparison of neuropsychological test performance and neuropsychiatric symptoms between right-handed individuals and non-right-handed individuals with behavioural variant frontotemporal dementia (*n* = 1132)

	Non-right-handed		Right-handed		*P*
	Mean	SD	Mean	SD	
**Neuropsychological tests**					
Animal fluency	10.60	6.34	9.90	6.39	0.308
Vegetable fluency	6.71	4.21	6.29	4.28	0.367
F + L phonemic fluency	15.04	9.66	13.83	9.53	0.542
TMT-A	58.15	39.27	62.94	38.16	0.268
TMT-B	149.81	85.37	173.85	95.11	**0**.**028**
MMSE^a^	22.06	6.75	20.78	7.87	0.117
Logical Memory Immediate^a^	6.26	4.94	6.20	4.84	0.916
Logical Memory Delayed^a^	4.00	4.43	4.85	4.67	0.143
Digit Span Forward^a^	7.00	2.53	6.47	2.61	0.094
Digit Span Backward^a^	4.54	2.30	4.02	2.49	0.083
Boston Naming Test^a^	20.42	7.88	20.09	8.59	0.748
Digit Symbol^a^	30.39	14.95	29.38	14.86	0.605
MoCA^b^	19.10	7.21	17.43	6.59	0.244
Craft Story Immediate^b^	10.42	5.21	9.21	5.69	0.276
Craft Story Delayed^b^	8.69	6.00	7.51	5.97	0.353
Number Span Forward^b^	7.31	2.13	6.33	2.40	**0**.**036**
Number Span Backward^b^	5.15	1.97	4.07	2.50	**0**.**015**
Multilingual Naming Test^b^	25.73	5.39	24.01	7.74	0.157
Copy of Benson Figure^b^	13.92	3.91	13.83	3.27	0.906
Benson Figure Delayed Recall^b^	6.50	3.88	6.70	4.67	0.808
**Neuropsychiatric symptoms**					
Delusions	0.17	0.57	0.28	0.70	0.048
Hallucinations	0.09	0.37	0.12	0.46	0.425
Agitation/aggression	0.78	0.97	0.87	0.99	0.341
Depression/dysphoria	0.51	0.79	0.61	0.85	0.176
Anxiety	0.77	0.97	0.80	0.97	0.769
Elation/euphoria	0.26	0.60	0.45	0.84	**0**.**002**
Apathy/indifference	1.59	1.09	1.59	1.09	0.995
Disinhibition	1.17	1.13	1.27	1.11	0.345
Irritability/lability	0.96	1.07	0.97	1.08	0.884
Motor disturbance	1.07	1.10	1.10	1.15	0.754
Nighttime behaviours	0.74	1.02	0.76	1.01	0.851
Appetite/eating	0.98	1.09	1.14	1.10	0.146

Numbers are raw scores in neuropsychological tests and the severity rating of Neuropsychiatric Inventory Questionnaire. Nominally significant *P*-values are bolded. MMSE, Mini-Mental State Examination; MoCA, Montreal Cognitive Assessment.

a UDS version 2 tests.

b UDS version 3 tests.

The results indicate that non-right-handed individuals performed slightly better than right-handers in 12 out of 20 neuropsychological tests in the CU group (*P*’s < 0.016, Cohen’s *d*’s ranging from 0.07 to 0.18) in unadjusted analyses. Non-right-handers performed better than right-handers also in some neuropsychological tests in all neurodegenerative disease groups with small to moderate effect sizes (*P’s* < 0.036, *d*'s ranging from 0.08 to 0.44). The most consistent pattern was seen in TMT-A: non-right-handers were ∼3 s [*t*(2186.4) = −7.7318, *P* = 1.606 × 10^−14^, *d* = 0.18) faster than right-handers in the CU group, and the difference was ∼6 s in those with Alzheimer’s disease [*t*(907.63) = −3.8545, *P* = 0.0001242, *d* = 0.15) after adjusting for multiple comparisons. Similarly, non-right-handers performed ∼5 s faster in TMT-A in those with bvFTD (*d* = 0.13) and ∼7 s (*d* = 0.13) in those with DLB, although these differences were not significant [bvFTD *t*(117.8) = −1.1135, *P* = 0.2677; DLB *t*(52.886) = −0.96025, *P* = 0.3413]. There was no diagnosis × handedness interaction in TMT-A [*F*(2,26389) = 1.515, *P* = 0.169].

After correcting for multiple comparisons, non-right-handers also performed better than right-handers in phonemic fluency, TMT-B, Boston Naming Test and Number Span Forwards and Backwards in the CU group (Table 2). No significant differences in these tests were observed in Alzheimer’s disease, DLB and bvFTD (Tables 3–5).

Differences in neuropsychiatric symptom ratings were scarcer, with non-right-handed individuals with Alzheimer’s disease having more anxiety symptoms than right-handers [*t*(1099.5) = 2.4752, *P* = 0.01347, *d* = 0.09]. In contrast, right-handed individuals with bvFTD had more severe euphoria than non-right-handers [*t*(181.74) = −3.1276, *P* = 0.002053, *d* = 0.23]. Right-handed individuals with DLB had more severe euphoria [*t*(135.88] = −2.2924, *P* = 0.02342, *d* = 0.17] and apathy [*t*(75.064) = −2.1472, *P* = 0.03501, *d* = 0.27]. However, none of the handedness differences in neuropsychiatric symptoms were evident after correcting for multiple comparisons (Tables 3–5).

Multiple regression analyses were conducted to investigate whether handedness showed associations with neuropsychological function and neuropsychiatric symptoms after adjusting for age, gender and education (Table 6; see Supplementary Tables 2–5 for all coefficients). After adjusting for these covariates and multiple comparisons, only an association between handedness and TMT-A performance (*β* = 0.033, adjusted *P* < 0.001) in the CU group remained. This difference amounted to right-handed individuals performing 1.76 (SE 0.37) s slower than the non-right-handed (Table 6).

**Table 6:** Multiple regression analyses of handedness on neuropsychological and neuropsychiatric scores with age, education and sex as covariates

	CU		Alzheimer’s disease		DLB		bvFTD	
	*β*	*P*	*β*	*P*	*β*	*P*	*β*	*P*
Animal fluency	−0.002	0.732	−0.001	0.913	−0.021	0.619	−0.023	0.486
Vegetable fluency	−0.001	0.885	0.006	0.535	0.002	0.965	−0.033	0.335
F + L phonemic fluency	−0.041	0.002	0.011	0.635	0.008	0.930	−0.030	0.659
TMT-A	**0.033**	**0.000**	0.034	0.002	0.039	0.392	0.034	0.335
TMT-B	0.018	0.007	0.008	0.506	−0.058	0.318	0.076	0.066
MMSE^a^	−0.008	0.373	−0.014	0.200	0.029	0.534	−0.040	0.270
Logical Memory Immediate^a^	−0.001	0.888	−0.011	0.364	0.036	0.468	0.002	0.952
Logical Memory Delayed^a^	0.002	0.850	0.005	0.688	0.020	0.690	0.065	0.107
Digit Span Forward^a^	−0.015	0.105	−0.012	0.282	0.053	0.291	−0.048	0.211
Digit Span Backward^a^	−0.013	0.140	−0.015	0.171	0.024	0.631	−0.044	0.253
Boston Naming Test^a^	−0.018	0.034	−0.011	0.328	−0.031	0.503	0.003	0.617
Digit Symbol^a^	0.008	0.322	−0.004	0.760	0.033	0.588	−0.021	0.617
MoCA^b^	0.006	0.601	−0.003	0.880	−0.056	0.504	−0.073	0.246
Craft Story Immediate^b^	0.024	0.069	0.024	0.306	0.012	0.891	−0.064	0.345
Craft Story Delayed^b^	0.020	0.136	0.027	0.256	−0.021	0.812	−0.064	0.354
Number Span Forward^b^	−0.039	0.003	−0.033	0.155	0.009	0.916	−0.121	0.062
Number Span Backward^b^	−0.034	0.011	−0.009	0.702	0.051	0.556	−0.138	0.035
Multilingual Naming Test^b^	−0.015	0.262	−0.015	0.535	−0.033	0.697	−0.052	0.425
Copy of Benson Figure^b^	0.033	0.014	0.014	0.543	−0.156	0.084	−0.009	0.896
Benson Figure Delayed Recall^b^	0.027	0.041	0.040	0.097	−0.288	0.002	0.013	0.840
Delusions	−0.014	0.069	−0.001	0.922	0.004	0.923	0.046	0.129
Hallucinations	0.001	0.929	0.009	0.381	−0.042	0.297	0.018	0.545
Agitation/aggression	0.007	0.373	0.003	0.770	0.006	0.885	0.030	0.322
Depression/dysphoria	0.009	0.231	−0.002	0.814	0.029	0.483	0.037	0.233
Anxiety	−0.008	0.296	−0.025	0.012	−0.024	0.550	0.009	0.768
Elation/euphoria	−0.004	0.649	−0.005	0.598	0.045	0.269	0.072	0.018
Apathy/indifference	0.011	0.148	−0.004	0.669	0.085	0.037	0.003	0.912
Disinhibition	0.008	0.325	0.002	0.843	0.044	0.281	0.029	0.347
Irritability/lability	0.005	0.533	0.001	0.899	0.003	0.935	0.006	0.852
Motor disturbance	−0.007	0.368	0.014	0.154	0.059	0.147	0.010	0.740
Nighttime behaviours	0.003	0.686	−0.013	0.200	0.014	0.733	0.009	0.770
Appetite/eating	0.002	0.811	−0.007	0.466	0.033	0.415	0.044	0.143

*β* values are standardized regression coefficients. Right-handedness is used as the reference. Bolded values are significant after Bonferroni–Holm adjustment for multiple comparisons. MMSE, Mini Mental-State Examination; MoCA, Montreal Cognitive Assessment.

a UDS version 2 tests.

b UDS version 3 tests.

Finally, to test whether collapsing left-handed and ambidextrous individuals affected our results, we performed the same multiple regression analyses in left- and right-handed individuals, excluding ambidextrous individuals (Supplementary Table 6). After adjusting for age, gender, education and multiple comparisons, the association between right-handedness and worse performance in TMT-A remained in the CU group. In addition, right-handers had poorer performance in Number Span Forward compared with left-handers in the CU group (Supplementary Table 6). These differences amounted to 2.38 (SE 0.42) s and 0.44 (SE 0.11) correct trials in favour of left-handed individuals, respectively. No statistically significant relationships were found in Alzheimer’s disease, DLB or bvFTD groups between handedness and clinical variables when excluding ambidextrous individuals (Supplementary Table 6). There were also no associations between ambidexterity and clinical variables in CU and Alzheimer’s disease groups: two groups with adequate sample sizes for such analyses (Supplementary Table 7).

## Discussion

To date, no large-scale studies of the relationship between handedness, neuropsychological performance and neuropsychiatric symptoms have been carried out in neurodegenerative diseases. This study set out to fill this gap.

The prevalence of left-handedness (7.8%) in our study was slightly lower than reported estimate of ∼10% in the most recent meta-analysis,^34^ but it should be noted that population-based studies including older cohorts suggests even low as 6% prevalence of left-handedness.^35^ We also detected the expected gender difference of higher prevalence of left-handedness in men compared with women.^36^ Taken together, our sample is reasonably representative with regard to handedness. We also found a slightly higher prevalence of left-handedness in those with bvFTD and DLB compared with those with Alzheimer’s disease or CU individuals. However, these differences were relatively small and should be considered with caution owing to the self-reported measure of handedness.

We found that after adjusting for gender, education, age and multiple comparisons, only slightly slower performance in TMT-A was found in CU right-handed individuals compared with the non-right-handed. Similar effect size was observed in Alzheimer’s disease, bvFTD and DLB groups, but the differences were non-significant in these smaller groups. When excluding ambidextrous individuals, right-handers performed worse than left-handers in TMT-A and Number Span Forward in the CU group, whereas no differences emerged in neurodegenerative disease groups.

Our findings relating to TMT-A are somewhat different to previous studies. Beratis *et al*.^37^ found left-handers to perform better in TMT-B but not in TMT-A, whereas Bracken *et al*.^38^ found right-handers to perform faster in both pen-and-paper and digital versions of TMT. While some previous studies suggest the handedness difference in TMT performance to reflect the contribution of the right hemisphere to complex cognitive functions, we are more cautious as we found differences only in TMT-A and in a specific span task in cognitively healthy individuals but not in multiple other tasks tapping into the same cognitive abilities.

If handedness was associated with domains usually thought to underlie TMT-A performance, such as visual tracking, processing speed and working memory,^39^ we could have expected to observe associations between handedness and the Benson figure task, Digit Symbol and Digit/Number Span tasks. This, however, was not the case, as only Number Span Forwards remained significant in the left- versus right-handedness comparison and only in the CU group. In span tasks, handedness differences were not observed in Digit Span Forwards (assessing the same construct and *n* > 6000 greater than Number Span Forwards) nor in Digit/Number Span Backward conditions, which are thought to rely more heavily on working memory compared with Forward conditions.^40^

Furthermore, the TMT instructions require the participant to keep their pencil on the paper, and this can lead to the performing hand to cover the sought targets.^38^ Thus, individual elements of the TMT are completed faster or slower based on the hand used,^41^ and subjects may have adapted their performance in Part B. Owing to the lack of differences in other tasks thought to correlate strongly with TMT performance and the inconsistent handedness differences in the previous literature, we propose that the slower performance by right-handed individuals in TMT-A may be related to the layout of the test rather than a meaningful difference in cognitive functioning.

The general finding of lack of handedness differences in neuropsychological performance is in line with those reported previously in Alzheimer’s disease, schizophrenia and semantic variant primary progressive aphasia.^4,7,8^ Considering our findings in TMT, it should be appreciated that the standardized coefficients for TMT were not markedly larger than those in a previous schizophrenia study, where the corresponding associations were non-significant.^7^ In our study, a raw difference of 2.9 s between non-right-handers and right-handers was significant (when using both NACC^42^ and other normative data^43^ as reference, the difference was ∼0.25 SD), whereas the handedness difference of 2 s (equivalent to 0.23 SD^43^) in the control group of the schizophrenia study^7^ was not. Thus, comparison of results from different studies should be considered in relation to sample size. Other aspect is to consider the clinical significance of the difference. To this end, we note that the observed effect size in the TMT is rather small. Sample size should be considered also when looking at different groups in our study: the advantage of the non-right-handers in TMT-A was even larger in the Alzheimer’s disease, bvFTD and DLB groups than in CU, but our formal interaction testing indicated that the association of handedness with TMT-A was similar in all these groups. This suggests that the faster performance of non-right-handers is evident universally in aging and supports the view that handedness effect in TMT-A is likely to arise from test stimuli that are differently visible for those who hold the pencil in left hand versus right hand.

We found no associations between handedness and neuropsychiatric symptoms despite increased prevalence of non-right-handedness in major psychiatric illnesses.^5,6^ Psychosis in Alzheimer’s disease is perhaps the most extensively researched neuropsychiatric syndrome in terms of genetics and neurobiology out of the three neurodegenerative diseases studied here. Recent results have indicated mainly no or negative genetic association between schizophrenia or bipolar disorder and psychosis in Alzheimer’s disease.^44^ Thus, whatever the role of excessive non-right-handedness in schizophrenia and bipolar disorder may be, the underlying genetics have not previously overlapped with those of increased risk for psychosis in Alzheimer’s disease (notwithstanding some single-nucleotide polymorphisms).^44^ More broadly, neuropsychiatric symptoms are known to be multifactorial, and psychosocial factors may contribute more to these symptoms compared with neuropsychological deficits. Similarly, the aetiology of depression in CU individuals is known to be complex, and no association was found between handedness and depression in a meta-analysis of studies concerning CU subjects.^45^

Limitations of the study include the simplicity of the handedness measure. Also, we had no information about forced right-handedness that is more common in older cohorts.^46^ Less data were available on UDS version 3 tests, and it is possible that we lacked power to detect differences in these tests. However, such possibly undetected differences would have to be small in most cases. We only studied participants with diagnosed dementia, and by definition, any possible role of handedness in preclinical states is outside the scope of this paper. It should also be acknowledged that the CU group is more highly educated than the general population and relatively healthy in terms of somatic or psychiatric conditions.^18^

Strengths of the study include a large sample size and adjustment for common factors associated with cognition and neuropsychiatric symptoms. The participants in our study did not have mixed dementia diagnoses. Our interest was in neurodegenerative diseases, but data also provide a reference for handedness differences in CU older adults. We used a relatively liberal adjustment method for multiple comparisons, reducing the likelihood of rejecting true positive associations.

In general, we found no differences in neuropsychiatric symptoms or neuropsychological test performance in right-handed and non-right-handed individuals with or without neurodegenerative disease. The observed difference in the TMT is likely to reflect test properties rather than differences in underlying cognitive functions. Our findings suggest that hand preference does not play a significant role in neuropsychological or neuropsychiatric phenotypes in common neurodegenerative diseases.

## Supplementary Material

fcad137_Supplementary_Data

## Data Availability

This study used NACC data from the March 2022 data freeze, which is freely available to interested researchers.

## Abbreviations

bvFTD =: behavioural variant frontotemporal dementia
CDR =: Clinical Dementia Rating
CU =: cognitively unimpaired
DLB =: dementia with Lewy bodies
NACC =: National Alzheimer’s Coordinating Center
SE =: standard error
TMT =: Trail Making Test
UDS =: Uniform Data Set
